# Nitidine Chloride Triggers Autophagy and Apoptosis of Ovarian Cancer Cells through Akt/mTOR Signaling Pathway

**DOI:** 10.2174/1381612829666230614154847

**Published:** 2023-07-18

**Authors:** Chaoqun Lian, Yinlong Huang, Ping Hu, Yuncheng Cao, Zhiqiang Zhang, Fan Feng, Jing Zhang

**Affiliations:** 1 Research Center of Clinical Laboratory Science, Bengbu Medical College, Bengbu, 233030, China;; 2 Department of Genetics, School of Life Sciences, Bengbu Medical College, Bengbu, 233030, China;; 3 School of Biology and Food Engineering, Suzhou University, Anhui, 234000, China

**Keywords:** Ovarian cancer, nitidine chloride, cell autophagy, cell proliferation, cell apoptosis, Akt/mTOR pathway

## Abstract

**Objective:**

Ovarian cancer (OC) is the eighth most common cancer with high mortality in women worldwide. Currently, compounds derived from Chinese herbal medicine have provided a new angle for OC treatment.

**Methods:**

In this study, the cell proliferation and migration of ovarian cancer A2780/SKOV3 cells were inhibited after being treated with nitidine chloride (NC) by using MTT and Wound-Healing Assay. Flow cytometry analysis indicated NC-induced apoptosis of ovarian cancer cells, and AO and MDC staining showed that NC treatment induced the appearance of autophagosomes and autophagic lysosomes in ovarian cancer cells.

**Results:**

Through the autophagy inhibition experiment of chloroquine, it was proved that NC significantly further promoted apoptosis in ovarian cancer cells. Furthermore, NC proved that it could significantly decrease the expression of autophagy-related genes such as Akt, mTOR, P85 S6K, P70 S6K, and 4E-BP1.

**Conclusion:**

Therefore, we suggest that NC could trigger autophagy and apoptosis of ovarian cancer cells through Akt/mTOR signaling pathway, and NC may potentially be a target for chemotherapy against ovarian cancer.

## 1 INTRODUCTION

Ovarian cancer (OC) is the eighth most common cancer in women worldwide, and also one of the leading gynecologic diseases with high mortality worldwide [1]. Due to the insidious onset of ovarian cancer and a lack of effective early diagnostic indicators, ovarian cancer is often found in the advanced stage, which leads to low survival rates [2]. According to the latest statistics, there are about 300,000 female ovarian cancer patients in the world each year, with 185,000 deaths and a mortality rate of 0.45% [3]. The pathogenic mechanism of ovarian cancer is still unclear, which is generally considered to be the result of heredity and environment. Cytoreductive surgery is an effective method for ovarian cancer treatment, but its efficacy for patients with advanced ovarian cancer is still limited. At present, platinum-based combination chemotherapy is the first-line treatment for advanced ovarian cancer [4]. However, in the initial stage of chemotherapy, 20% of patients do not respond to platinum chemotherapy, and even up to 75% of ovarian cancer patients have a relapse. At the same time, patients receiving treatment after relapse often develop resistance to chemotherapy drugs [5, 6]. Therefore, identifying the sensitivity of patients to chemotherapy drugs and screening effective combined chemotherapy drugs co-effectively improve the chemotherapy efficacy of ovarian cancer.

With the continuous development of chemotherapy drugs, combined chemotherapy for ovarian cancer is still the main method. In recent years, some Chinese herbal medicine ingredients have been found to have anti-cancer effects of inhibiting the proliferation of cancer cells and inducing apoptosis, thus improving the sensitivity of patients to chemotherapy drugs *in vivo* and/or *in vitro*, such as genistein, curcumin, phytosterols, ginsenoside, quercetin, *etc*. [7, 8]. The mechanism of action of these Chinese herbal ingredients includes influencing gene expression, anti-oxidation, inhibiting the activity of key enzymes in cell metabolism, and inhibiting tumor angiogenesis [9]. The bioactive components of traditional Chinese medicine, as adjuvant therapy of chemotherapy, have broad clinical application prospects. Generally, traditional Chinese medicines (TCMs) achieve their therapeutic effects through synergistic interactions *via* multi-components and multi-targets [10]. Natural products mainly come from plants, microorganisms, and animals, including quinones, phenylpropanoids, terpenoids, flavonoids, steroids, polysaccharides, alkaloids, and so on [7]. Among them, alkaloids are very important natural organic compounds with great medicinal potential and value. Since the discovery of the first alkaloid morphine in 1806, about 130,000 known alkaloids have been discovered [11]. A large number of studies have shown that alkaloids have extensive pharmacological effects, such as anti-cancer, antioxidation, anti-inflammation, and immune regulation [12]. At present, there are more than 100 kinds of alkaloids that have been used in clinical treatment, such as berberine, quinine, scopolamine, hyoscyamine, cocaine, anisodamine, and ephedrine [13]. The research on alkaloids have always been one of the important research fields in the fields of natural product chemistry, pharmacy, and pharmaceutical chemistry, which has constantly aroused the interest of scientists and lasted for a long time.

Nitidine chloride (NC), a quaternary ammonium benzophenanthridine alkaloid, was initially isolated from the roots of *Zanthoxylum nitidum* (Roxb.) DC. by Arthur *et al*., in 1959, and then was successively found in many other medicinal plants [14]. Due to its relatively high content in *Zanthoxylum nitidum*, currently, nitidine chloride is mainly extracted from the roots or stems of *Zanthoxylum nitidum* which is a common traditional Chinese medicine [15]. *Zanthoxylum nitidum* has many traditional effects, such as promoting blood circulation and removing blood stasis, detoxication and detumescence, and expelling wind and dredging meridian [16]. In ancient China, *Zanthoxylum nitidum* was usually used as the main component of prescriptions to treat toothache, trauma, and gastrointestinal diseases [17]. With the continuous deepening and development of modern pharmacological research, NC showed a wide range of pharmacological activities, including anti-inflammation, anti-tumor, anti-colitis, anti-osteoporosis, anti-malaria, and so on [18]. In the field of anti-tumor, it is found that NC can affect and regulate the growth of breast cancer, lung cancer, liver cancer, gastric cancer, *etc*. [19], but research in ovarian cancer is still relatively scarce. The existing literature mainly focuses on NC's influence on the proliferation of ovarian cancer cells through ERK, AKT, and Fas pathways [20, 21], but few researchers have found out whether NC affects autophagic apoptosis of ovarian cancer cells through regulating signal pathways.

Therefore, this study aimed to determine the effects of NC on apoptosis and autophagy in ovarian cells. Effects of NC on proliferation and migration of ovarian cancer cells were detected by using MTT and cell scratch test. Flow cytometry was used to detect the effect of NC on apoptosis and autophagy of ovarian cancer cells, and the Akt/mTOR signaling pathway in ovarian cancer was evaluated by using western blot experiment. All these results suggest that NC can effectively promote autophagic apoptosis of ovarian cancer cells through Akt/mTOR signaling pathway, which provides a theoretical basis for further research on NC's inhibition of tumor cell growth.

## 2 MATERIALS AND METHODS

### 2.1 Cell Culture and Chemicals

Ovarian cancer cell lines SKOV3 and A2780 were purchased from the Cell Bank of the Chinese Academy of Sciences (Shanghai, China) and KeyGEN BioTECH Co., Ltd. (Jiangsu, China). They were maintained in DMEM (high sugar) medium and McCOY's 5A medium with 10% FBS and 1% penicillin-streptomycin (37^o^C, 5% CO_2_).

### 2.2 Reagents and Antibodies

Nitidine chloride (NC, CAS no. 13063-04-2) was ordered from Tauto Biotech Co., Ltd. (Shanghai, China). 3-[4,5-Dimethylthiazol-2-yl]-2,5-diphenyltetrazolium bromide (MTT) assay was purchased from Beyotime Institute of Biotechnology (Shanghai, China), FITC Annexin V Apoptosis Detection Kit I(#556547) was purchased from BD Biosciences (Franklin Lakes, NJ, USA). Chloroquine (CQ) was ordered from Selleck. Monodansylcadaverine (MDC) or acridine orange (AO) were purchased from Solarbio Science & Technology Co., Ltd. (Beijing, China). The primary antibodies were provided by Cell Signaling Technology (USA), including LC3A/B (D3U4C, #12741, 1:1000), SQSTM1/p62 (D5E2, #8025, 1:1000), Akt (C67E7, #4691, 1:1000), Phospho-Akt (Thr308, D25E6, #13038, 1:1000), mTOR (7C10, #2983, 1:1000), Phospho-mTOR (Ser2448, D9C2, #5536, 1:1000), p70 S6 Kinase (49D7, #2708, 1:1000), Phospho-p70 S6 Kinase (Thr389, 108D2, #9234, 1:1000), 4E-BP1 (53H11, #9644, 1:1000), Phospho-4E-BP1 (Thr37/46, 236B4, #2855, 1:1000), β-Actin (13E5, #4970, 1:5000).

### 2.3 Cell Proliferation Assay

To better observe the inhibitory effect of NC on tumor cell proliferation, we planted ovarian cancer cells in a 96-well plate at a density of 3000 cells/well. After overnight culture, the medium was removed and replaced with different concentrations of the NC for 24 hours, 48 hours, and 72 hours. 20 μL MTT was added to each well and 150 μL of DMSO was used to dissolve the crystals after incubated 4 hours. The absorbance at 570 nm was measured with a multi-functional microplate reader (BioTek, cytation3, USA).

### 2.4 Wound-healing Assay

On the back of the six-well plate, evenly spaced lines were drawn with a marker, and the interval between each line was 0.5-1.0 cm. The cells were resuspended and planted in a six-well plate at an appropriate density to ensure that they could cover the bottom of the plate after overnight incubation. The pipette tip was used to draw a uniform straight line perpendicular to the six-well plate. After the cells were washed with PBS, serum-free medium and drugs were added and photographed at 0 hours and 24 hours, respectively, and then the cells were placed in an incubator.

### 2.5 Cell Apoptosis Assay

Ovarian cancer cells were resuspended and implanted in a 6-well plate until adhered and treated with drugs for 48 hours. The cells were digested with trypsin without Ethylenediaminetetaacetic acid (EDTA), washed with pre-cooled PBS, and centrifuged at 1200 r/min for 5 minutes to collect the cells. The cells were resuspended to a density of 1×10^5^ cells/cm^3^ with binding buffer and added to the flow tube. Then the cells were incubated with 5 μL FITC Annexin V and 5 μL PI in the dark (25°C) for 15 minutes, and then 400 μL binding buffer (1x) was added. The samples were placed in ice and measured by the flow cytometer within 1 hour.

### 2.6 Detection of Autophagy by AO and MDC Staining

Cells were planted on Petri dishes at a density of 6×10^4^ cells/ cm^3^ and cultured overnight. The cells are washed once with washing buffer, stained with monodansylcadaverine (MDC) for 45 minutes, and acridine orange (AO) for 15 minutes. After washing twice with PBS, serum-free medium was added, and the laser confocal microscope was used to observe and take pictures.

### 2.7 Western Blot

The ovarian cancer cells were planted on a six-well plate, and treated with NC, DMSO or CQ for 48 hours. Then cells were washed with PBS thrice and added RIPA lysis solution. The total protein was collected with cell scrapers and centrifuged at 13000 r/min for 30 min. The concentration of protein solution was determined by BCA protein assay, and the quantitative 60-80 μg protein was separated by 10% SDS-PAGE. After being transferred to the PVDF membrane, it was sealed with skimmed milk at room temperature for 2 hours, and incubated in the primary antibody overnight. Then the PVDF membrane was washed with Tris-buffered saline solution containing Tween 20 and then incubated in the secondary antibody for two hours. The chemiluminescent HRP substrate (Millipore, USA) was mixed and evenly smeared on the surface of the membrane. The results were visualized by Tanon 5200 Detection System (Shanghai, China) and analyzed by ImageJ software.

### 2.8 Statistical Analysis

The data are shown as mean ± standard deviation. GraphPad Prism 6.0 software used ANOVA to evaluate statistical significance in multiple groups. A *p-value* less than 0.05 is considered statistically significant.

## 3 RESULTS

### 3.1 Nitidine Chloride Inhibited Cell Viability and Migration of Ovarian Cancer Cells

**Fig. (1):**
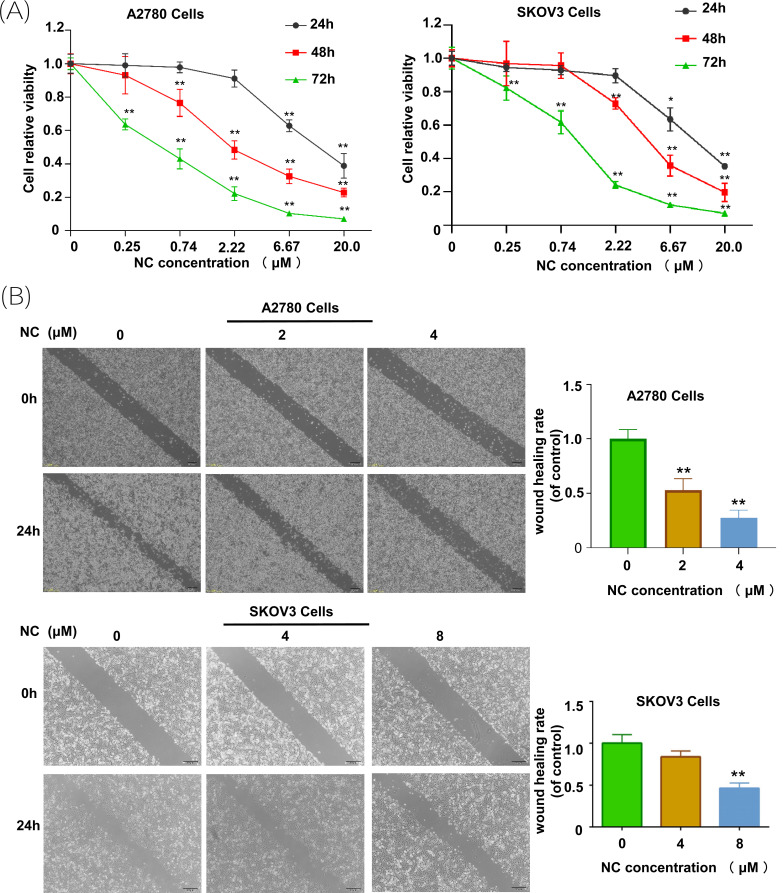
Nitidine chloride inhibited cell viability and migration of ovarian cancer cells. (**A**) Nitidine chloride inhibited cell viability of ovarian cancer A2780/SKOV3 cells. Cell viability was determined using MTT assays, and the calculation of IC_50_ was obtained by using Probit regression analysis in SPSS 18.0 software; (**B**) Nitidine chloride inhibited cell migration of ovarian cancer A2780/SKOV3 cells. (**p < 0.01, student’s *t*-tests).

To identify the role of nitramine chloride in ovarian cancer cells, MTT assays and wound healing assays were conducted in ovarian cancer cells. In MTT experiments, A2780 and SKOV3 cells were incubated with different concentrations of NC (0, 0.25, 0.74, 2.22, 6.67, 20.0 μM) for 24, 48, and 72 h, respectively. The results indicated that the NC could significantly inhibit the viability of ovarian cancer cells compared with the control group, and this effect was more obvious with increasing dose and time (Fig. **1A**). Almost 50% cell viability in A2780 and SKOV3 cells was suppressed by 2.831 μM and 4.839 μM NC after 48 h treatment, respectively. Therefore, 2 μM and 4 μM NC were chosen for A2780 cells and 4 μM and 8 μM NC were chosen for SKOV3 cells to conduct subsequent experiments. As shown in Fig. (**1B**), nitramine chloride can suppress ovarian cancer cell migration, and this inhibition has significant statistical significance when the concentration of NC exceeds the IC_50_ value.

### 3.2 Nitidine Chloride Promoted Cell Apoptosis of Ovarian Cancer Cells

**Fig. (2):**
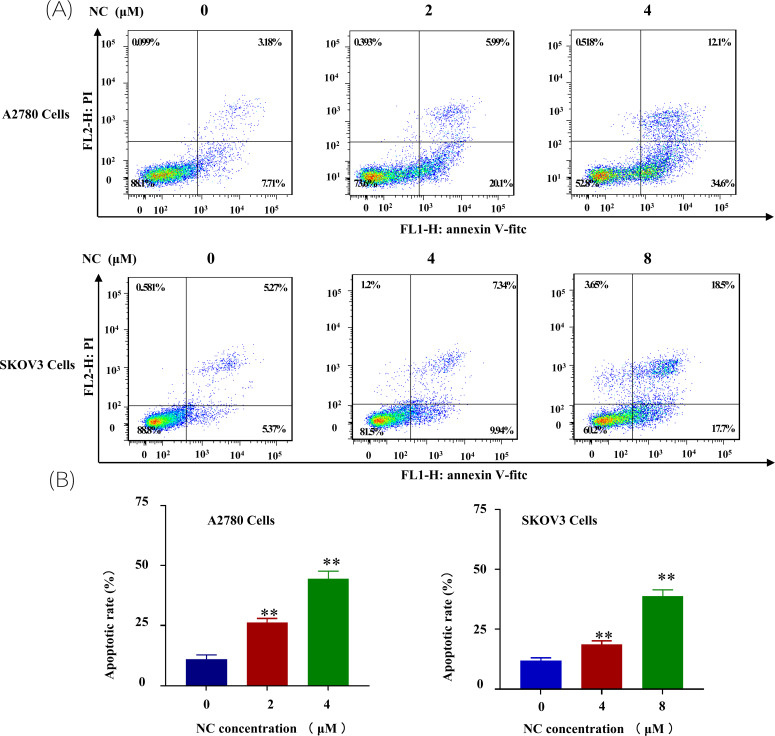
Nitidine chloride promoted cell apoptosis of ovarian cancer A2780/SKOV3 cells. (**A**) Apoptosis of ovarian cancer A2780 cells after nitidine chloride treatment; (**B**) Apoptosis of ovarian cancer SKOV3 cells after nitidine chloride treatment (**p < 0.01, student’s *t*-tests).

To investigate whether the NC could affect the apoptosis of ovarian cancer, the human ovarian cancer cells A2780 and SKOV3 were incubated with different concentrations NC for 48 h. Compared with the control group cells, Annexin V-FITC/PI assay showed that NC exposure induced cell apoptosis in both A2780 and SKOV3 cells. The results revealed that 2 μM and 4 μM NC increased the rate of early apoptosis from 10.89 ± 1.90% in the control group to 26.29 ± 1.72% and 44.4 ± 3.36% in A2780 cells, respectively (Fig. **2**). At the same time, the apoptosis rates of SKOV3 cells treated with NC were 18.7,sis in both A2780 and SKOV3 cells. The results revealed nitidine chloride could significantly stimulate the apoptosis of ovarian cancer cells.

### 3.3 Nitidine Chloride Induced the Appearance of Autophagosomes and Autophagic Lysosomes of Ovarian Cancer Cells

AO and MDC staining were used to observe the phenomenon of autophagy. MDC was an acidophilic stain, which was often used for autophagy staining and showing green fluorescence. As shown in Fig. (**3A**), compared with the control, A2780 and SKOV3 cells were treated with NC at 2 μM and 4 μM for 48 hours, and the number of cells stained with MDC increased significantly, and the green fluorescence showed dot aggregation, which indicated the aggregation of autophagosomes.

**Fig. (3):**
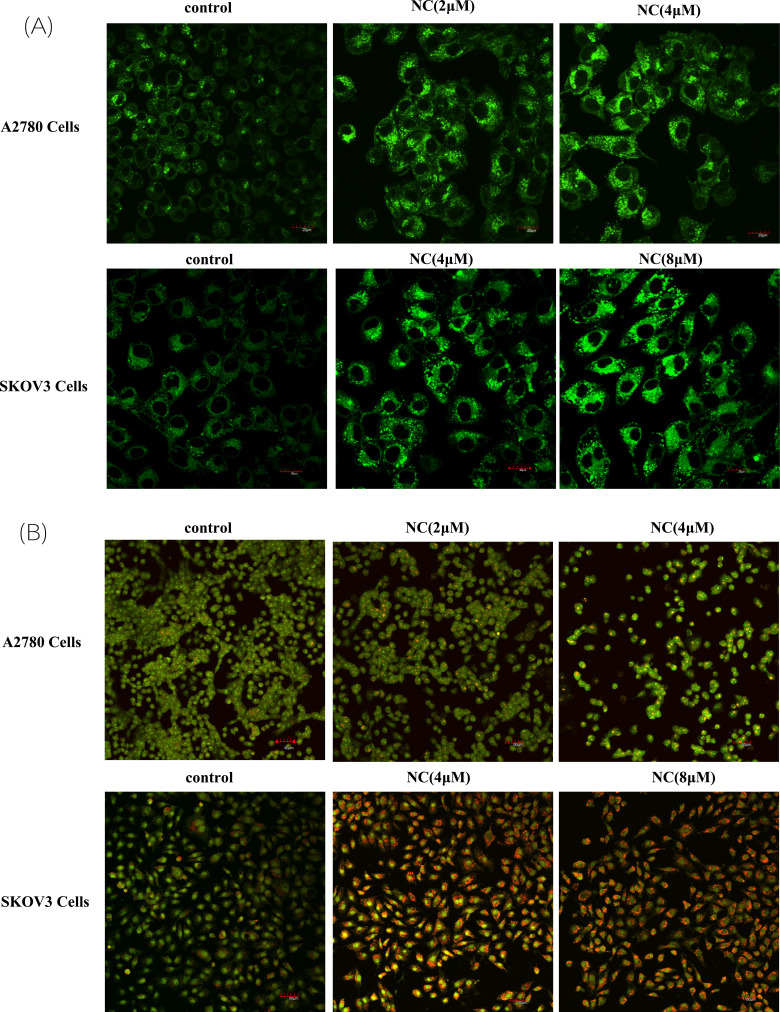
Nitidine chloride induced the appearance of autophagosomes and autophagic lysosomes of ovarian cancer cells. (**A**) Nitidine chloride induced the appearance of autophagosomes of ovarian cancer A2780/SKOV3 cells; (**B**) Nitidine chloride induced the appearance of autophagic lysosomes of ovarian cancer A2780/SKOV3 cells. The cells were observed and analyzed using a microscope with a ×10 objective.

AO could penetrate the cell membrane and insert into the nucleus to combine with DNA, and emit green fluorescence. It could also enter acidic organelles such as autophagic lysosomes, and emit red fluorescence in low pH environments. Compared with the control group, the NC-treated A2780 and SKOV3 cells showed red acid vesicles, indicating the aggregation of autophagic lysosomes, while the control group did not show obvious acid organelles (Fig. **3B**). These results suggested that NC could induce the formation of autophagy and autophagic lysosome.

### 3.4 Effect of Nitidine Chloride on the Autophagic Flow of Ovarian Cancer Cell

Autophagy was a dynamic process, and the increase in the number of the autophages may be due to the activation of autophagy or the damage of the downstream reaction of autophagy. Therefore, an LC3 turnover assay was used to evaluate the autophagic flow of cells. Fig. (**4**) showed that LC3-II/LC3-I in the group treated with CQ and NC was significantly higher than that in the group treated with CQ alone. It indicated that NC treatment promoted the generation of autophage in ovarian cancer cells. At the same time, the LC3-II/LC3-I ratio of the group treated with CQ and NC was also higher than that of the group treated with NC alone, indicating that NC did not inhibit the fusion of autophagy and lysosome.

### 3.5 Effects of Nitidine Chloride on Proliferation and Apoptosis of Ovarian Cancer Cells after Autophagy Inhibition

**Fig. (5):**
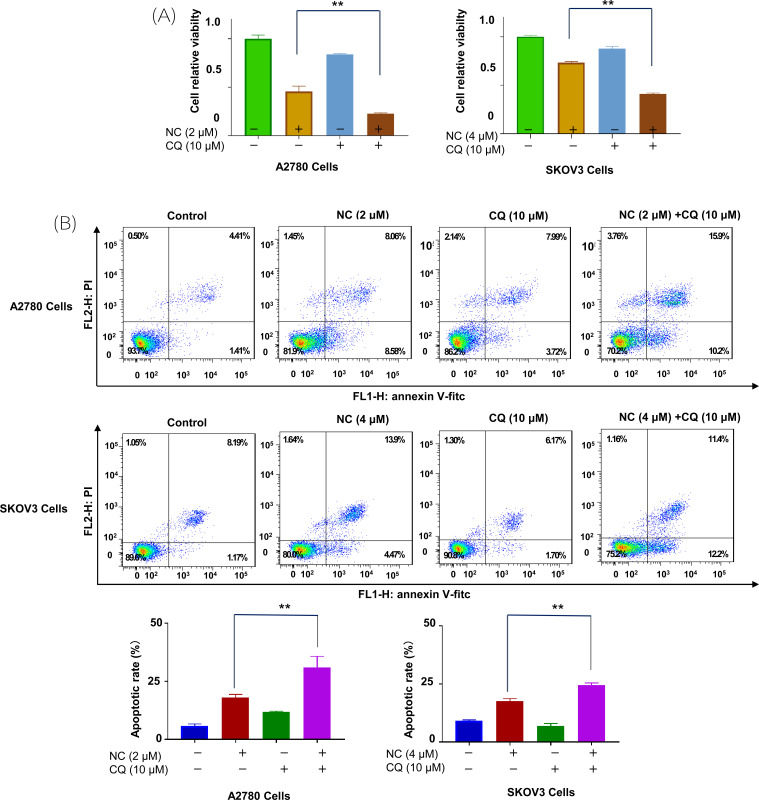
Effects of Nitidine chloride on proliferation and apoptosis of ovarian cancer cells after autophagy inhibition. (**A**) Effects of Nitidine chloride on the proliferation of ovarian cancer A2780/SKOV3 cells after autophagy inhibition. (**B**) Effects of Nitidine chloride on apoptosis of ovarian cancer A2780/SKOV3 cells after autophagy inhibition. **p < 0.01, SPSS.

CQ was a good inhibitor for autophagy research, when NC and CQ were combined, significant enhancement of NC-induced inhibition of cell viability was observed in A2780 and SKOV3 cells (Fig. **5A**). In addition, Annexin V/PI double staining also showed an increase in the proportion of apoptotic cells, indicating that autophagy may play a cytoprotective role in NC-induced cell death (Fig. **5B**).

### 3.6 Nitidine Chloride Inhibited Akt/mTOR Signaling Pathway of Ovarian Cancer Cells

The above experimental results showed that NC could inhibit the proliferation and induce apoptosis of ovarian cancer cells. Previous evidence indicated that the Akt/mTOR signaling pathway was associated with cell proliferation and apoptosis. However, it was not clear whether NC affected ovarian cancer cells through Akt/mTOR pathway. To test this possibility, the expression of key proteins in the Akt/mTOR pathway was detected after 48 h treatment by NC. As shown in Fig. (**6**), the levels of Akt, mTOR, P85 S6K, P70 S6K, and 4E-BP1 were reduced under NC treatment. In particular, compared with the control, NC treatment significantly inhibited the levels of p-Akt, p-mTOR, p-P85 S6K, p-P70 S6K, and p-4E-BP1. These findings indicated NC could inhibit the Akt/mTOR signaling pathway.

## 4 DISCUSSION

Ovarian cancer is one of the major gynecological diseases with high mortality worldwide. Although significant progress has been made in genomics, proteomics, and radiology, little progress has been made in translating these research advances into effective clinical treatment of ovarian cancer [22, 23]. Genetic mutation, other genetic factors, and chemical-induced carcinogenesis play a key role in the formation of ovarian tumors. More than 90% of ovarian malignant tumors are cancers, which are believed to originate from ovarian surface epithelium and/or fallopian tubes [24]. OC treated by radiochemotherapy schemes often caused serious adverse reactions, which seriously affect the quality of life and immune function of patients [25, 26]. Therefore, it is urgent to seek an alternative therapy that can improve the quality of life and immune function of patients. Several studies have recognized that traditional Chinese medicines have unique advantages in the treatment of malignant tumors by inhibiting the growth of cancer cells, enhancing human immunity, reducing cancer recurrence and metastasis, and slowing down disease progress [27-29].

*Zanthoxylum bungeanum* (ZB) belongs to *Rutaceae*, and its underground root is used as the medicinal part recorded in the Chinese Pharmacopoeia [30, 31]. ZB has excellent curative effects, such as treating toothache, stomachache, trauma, and rheumatoid arthritis. In daily life, ZB can be used as toothpaste and hand sanitizer. In a previous study, researchers focused on the chemical separation and activity evaluation of ZB [32, 33]. ZB extract showed good anti-inflammatory and antioxidant activities. Alkaloids, the main components of ZB, have attracted more and more attention [34]. Nitidine is a typical alkaloid in ZB, which has been found to have antifungal and anti-inflammatory activities, and its derivative NC has been found to have a wide range of anti-tumor activities [35-37], but research in ovarian cancer is still lacking. In this study, NC was proved to significantly inhibit the proliferation of ovarian cancer cells and promote the apoptosis of ovarian cancer cells, which is closely related to the autophagy process of ovarian cancer cells.

Cell autophagy is an important mechanism for cells to protect themselves under extreme conditions such as nutritional deficiency, drug intervention, and environmental stimulation, to promote cell survival [38]. Therefore, fine regulation of autophagy is extremely important for the direction of cell fate [39]. The involvement of autophagy in the regulation of malignant transformation of tumors and chemotherapy resistance is a research hotspot in recent years. A large number of studies show that autophagy is considered to hinder the occurrence of tumors in the early development of tumors, but after radiotherapy and chemotherapy, autophagy can timely remove and use harmful substances inside and outside cells to promote malignant transformation of tumors [40, 41], which may be the main reason why clinical cancer patients relapse and are difficult to treat after chemotherapy, Therefore, some scholars proposed to inhibit autophagy to improve the chemosensitivity of breast cancer cells [42]. Interestingly, NC can amplify the inhibition of proliferation and promotion of apoptosis of ovarian cancer cells after inhibition of autophagy of ovarian cancer cells by chloroquine, indicating that autophagy after NC treatment has a protective effect on ovarian cancer cells. A large number of studies have shown that autophagy is regulated by upstream PI3K/Akt/mTOR, LKBI/AMPK/mTOR, and other signal pathways [43-45]. This study demonstrated that NC can inhibit the expression and phosphorylation of Akt and mTOR proteins through WB experiments, indicating that NC can affect autophagy and promote apoptosis of ovarian cancer cells through Akt/mTOR pathway. Moreover, the downstream 4E-BP1 protein and its phosphorylated expression level were also significantly inhibited by NC, suggesting that NC mainly regulates the malignant phenotype of ovarian cancer cells through Akt/mTOR/4E-BP1 signaling pathway.

## CONCLUSION

To sum up, in this study, we found that NC can inhibit the proliferation of ovarian cancer cells and promote apoptosis of ovarian cancer cells. Combined with the autophagy inhibitor chloroquine, the results showed that NC could further induce apoptosis of ovarian cancer cells, indicating that inhibition of autophagy could improve the sensitivity of ovarian cancer cells to NC. Furthermore, NC has been proven to be able to regulate the malignant phenotype of ovarian cancer cells by regulating AKT/mTOR pathway. Accordingly, our results provide new insights into the role of NC as a potential therapeutic target to trigger the autophagic apoptosis of ovarian cancer.

## AUTHORS’ CONTRIBUTIONS

Revision for important intellectual content was contributed by FF, CQL, and JZ; Preparation of the manuscript was conducted by FF, PH, YCC, and YLH under the supervision of FF.

## Figures and Tables

**Fig. (4):**
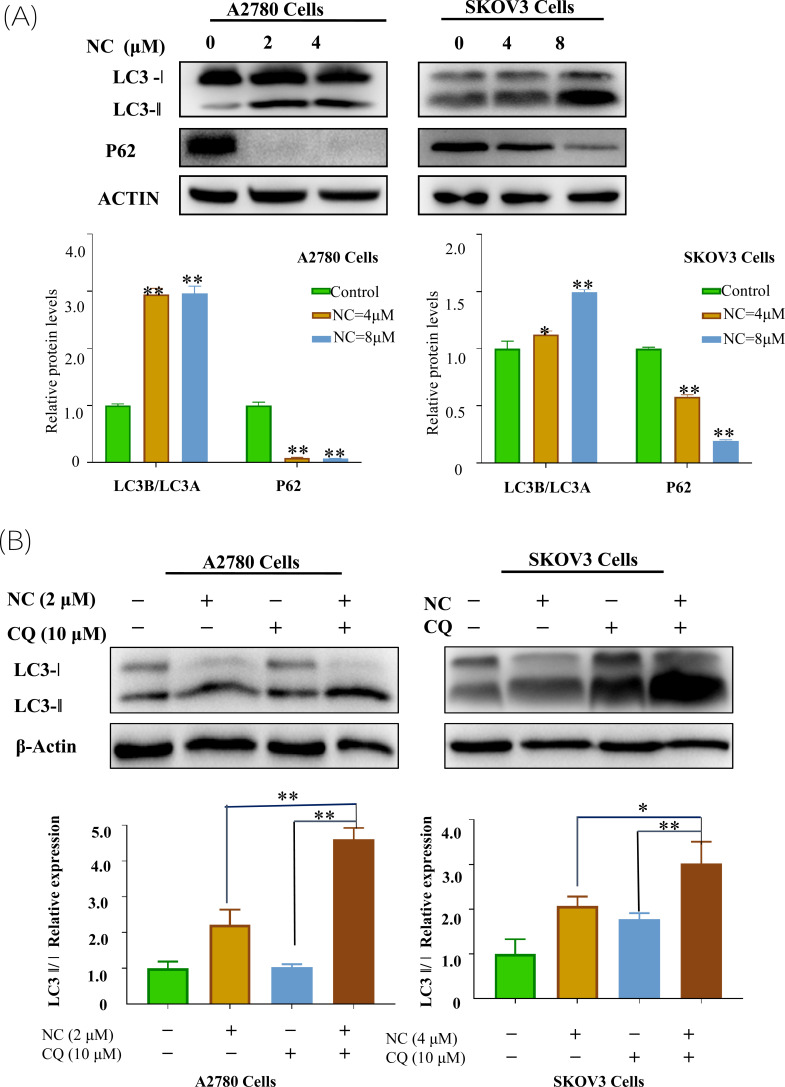
Effect of Nitidine chloride on autophagic flow of ovarian cancer cell. (**A**) Western blot analysis detected the expression levels of LC3-I, LC3-II, and P62 proteins after treatment of ovarian cancer A2780/SKOV3 cells with nitidine chloride. (**B**) Western blot analysis detected the expression level of proteins of LC3-I, and LC3-II after ovarian cancer A2780/SKOV3 cells treated with nitidine chloride, chloroquine, nitidine chloride and chloroquine, respectively. Student’s *t*-tests: *p < 0.05, **p < 0.01, SPSS.

**Fig. (6):**
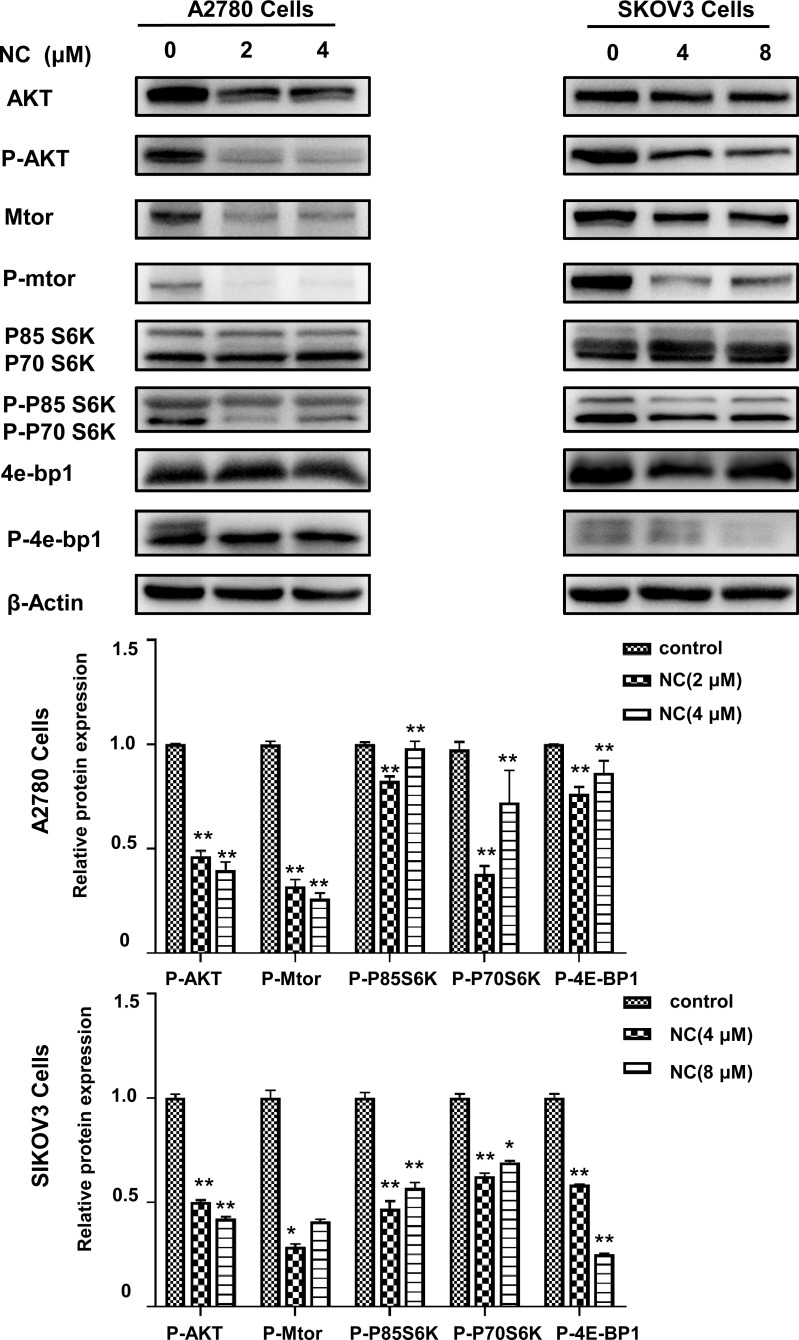
Nitidine chloride activated Akt/mTOR signaling pathway of ovarian cancer cells. Western blot analysis revealed the expression level of Akt, mTOR, P85 S6K, P70 S6K, 4E-BP1 and their phosphorylated proteins were lower in NC-treated ovarian cancer A2780/SKOV3 cells than that in the control group cells. Student’s *t*-tests: *p < 0.05, **p < 0.01, SPSS.

## Data Availability

The data and supportive information are available within the article.

## LIST OF ABBREVIATIONS

AO: Acridine Orange
MDC: Monodansylcadaverine
NC: Nitidine Chloride
OC: Ovarian Cancer
TCMs: Traditional Chinese Medicines
